# Risk of mycobacterial infections in a cohort of silicosis patients with autoimmune rheumatic diseases

**DOI:** 10.36416/1806-3756/e20240265

**Published:** 2024-11-16

**Authors:** Rafael Futoshi Mizutani, Ubiratan Paula Santos, Roberta Karla Barbosa Sales, Emily Figueiredo Neves Yuki, Elisa Maria Siqueira Lombardi, Lavinia Clara del Roio, Mario Terra-Filho

**Affiliations:** 1. Divisão de Pneumologia, Instituto do Coracao - InCor - Hospital das Clinicas -HCFMUSP - Faculdade de Medicina, Universidade de Sao Paulo, Sao Paulo (SP) Brasil.; 2. Divisão de Reumatologia, Hospital das Clinicas -HCFMUSP - Faculdade de Medicina, Universidade de Sao Paulo, Sao Paulo (SP) Brasil.

**Keywords:** Silicosis, Tuberculosis, Mycobacterium infections, nontuberculous, Arthritis, rheumatoid, Scleroderma, systemic, Lupus erythematosus, systemic

## Abstract

**Objective:**

To evaluate the incidence rates of mycobacterial infections in silicosis patients with systemic autoimmune rheumatic disease (ARD).

**Methods:**

This was a retrospective cohort of silicosis patients between January of 1999 and December of 2023. We compared the incidence of tuberculosis and nontuberculous mycobacterial disease (NTM) in patients with silicosis with and without ARD. We also compared the tuberculosis incidence in the overall cohort with general Brazilian population estimates.

**Results:**

The study comprised 369 silicosis patients, of whom 35 (9.5%) had ARD. Having ARD did not affect the cumulative incidence of mycobacterial diseases. The risk of tuberculosis was higher in the cohort when compared with that in the adult Brazilian male population (age-adjusted incidence rate ratio = 20.46; 95% CI 14.89-28.13).

**Conclusions:**

In this cohort of patients with silicosis, ARD was not associated with the incidence of mycobacterial diseases.

## INTRODUCTION

There is increasing evidence that silica exposure may trigger the pathogenesis of several systemic autoimmune rheumatic diseases (ARD), such as rheumatoid arthritis (RA),^1^ ^-^ ^3^ systemic sclerosis (SSc),^2^ ^-^ ^4^ systemic lupus erythematosus (SLE),^5^ and antineutrophil cytoplasmic antibody (ANCA)-associated vasculitis (AAV).^3^ ^,^ ^6^ Also, silica exposure and silicosis are known risk factors for developing mycobacterial infections.^1^ ^,^ ^7^ ^,^ ^8^ Furthermore, patients with ARD receiving systemic glucocorticoids, conventional synthetic disease-modifying antirheumatic drugs (csDMARD) and biologic/targeted synthetic treatment (b/tsDMARD) are at high risk of mycobacterial infections.^9^ ^,^ ^10^

Although there is biologic plausibility for a triple association of silicosis, ARD, and mycobacterial infections, there are only a few case reports in the literature exploring this topic,^11^ ^-^ ^14^ including an interim case series of our center.^15^

We hypothesized that patients with silicosis and ARD have a higher risk of mycobacterial infections than those without ARD. Therefore, we assessed the data of our silicosis patients to compare the incidence rate of mycobacterial infections between patients with silicosis and ARD and those without ARD, and to explore variables possibly associated with mycobacterial infections. We also compared the prevalence of ARD and the incidence of tuberculosis in this cohort with general population estimates in Brazil.

## METHODS

Consecutive patients with silicosis from a single tertiary care hospital with specialized services in occupational respiratory diseases in the city of São Paulo, Brazil, who had been followed between January of 1999 and December of 2023 were analyzed using a retrospective longitudinal cohort design.

### Case definition

Patients with silicosis and ARD were included in the case group. Silicosis was defined as the presence of compatible radiological features on HRCT^16^ in patients with occupational exposure to inhaled silica (for further information, see supplementary material, Table S1). Diagnoses of ARD were defined according to disease definitions stated by the 2010 American College of Rheumatology (ACR)/European Alliance of Associations for Rheumatology (EULAR) Classification Criteria for Rheumatoid Arthritis^17^; the 2013 ACR/EULAR Classification Criteria for Systemic Sclerosis^18^; the 2019 ACR/EULAR Classification Criteria for Systemic Lupus Erythematosus^19^; and the 2012 Chapel Hill Consensus Conference for Nomenclature of Vasculitides.^20^ Patients with symptoms, signs, and serological manifestations of ARD but with insufficient criteria for a specific disease were classified as having undifferentiated connective tissue disease (UCTD) as described by Mosca et al. in 2014.^21^

### Selection of control group

The remaining patients with silicosis were included in the control group. Patients using systemic glucocorticoids or synthetic immunosuppressive or biologic medications due to conditions other than ARD were excluded. Patients with a single evaluation in our service were also excluded.

### Mycobacterial infection definition

Tuberculosis was defined as a positive *Mycobacterium tuberculosis* culture or a positive molecular testing (nuclear amplification assays or Xpert® MTB/RIF assay) in sputum, bronchoalveolar lavage, biopsy fragment, or other biologic samples.^22^ Nontuberculous mycobacterial (NTM) disease was defined by the criteria of the 2007 American Thoracic Society/Infectious Disease Society of America Statement for Nontuberculous Mycobacterial Diseases.^23^

### Procedures

The study was approved by the University of São Paulo Research Ethics Committee (protocol number 5.360.480), which waived the requirement to obtain informed consent since data were collected from regular follow-up records and anonymized for analysis.

Patients were evaluated regarding their age at the start of follow-up; gender; tobacco consumption; occupational history of silica exposure; comorbidities; general and ARD-specific symptoms; serological tests and ARD-specific diagnostic evaluation; use of systemic corticosteroids; csDMARD or b/tsDMARD treatment; last available pulmonary function test before censoring, tuberculin skin test positivity (≥ 10 mm), tuberculosis or NTM diagnosis and/or treatment before follow-up; and positive AFB smear, mycobacterial culture or *M. tuberculosis* molecular assays.

We reviewed follow-up records of patients with silicosis since their first medical appointment in our center. Patients were considered to have had an event if they were diagnosed with tuberculosis or NTM during follow-up. Patients were censored after lung transplant, death for any cause, or at the last available appointment date if lost to follow-up. If none of these conditions were met, they were censored at December 31st, 2023.

### Statistical analysis

We compared characteristics of ARD and control groups using the Student’s t-test for normally distributed continuous variables, the Mann-Whitney test for non-normally distributed continuous variables, and the Fisher’s exact test for categorical variables.

Cumulative incidence rates of tuberculosis, NTM, and any mycobacterial disease in the ARD and control groups were analyzed using the Kaplan-Meier method and log-rank for comparisons.

Cox proportional hazards model was used to calculate unadjusted hazard risk of mycobacterial infections for the following variables: age at start of follow-up, tobacco consumption, diabetes, total occupational silica exposure time, FVC in percentage of predicted value (FVC%), FEV_1_%, any ARD diagnosis, use of systemic glucocorticoids for a period longer than six months, use of any csDMARD ever, use of any b/tsDMARD ever, prior tuberculosis, and latent tuberculosis treatment during follow-up.

Brazilian population estimates from 2001 to 2021 were retrieved using the Brazilian Institute of Geography and Statistics database.^24^ An age-adjusted incidence rate ratio (IRR) of tuberculosis in the overall cohort was calculated and compared with the tuberculosis incidence in the adult (> 20 years of age) Brazilian male population during the same time period using the Brazilian Case Registry Database,^25^ the national system for notification of diseases that is maintained by the Brazilian Ministry of Health. Because NTM notification is not mandatory in Brazil, there were no reliable Brazilian incidence estimates for NTM.^26^

We calculated the prevalence ratios (PR) of RA, SLE, and SSc and compared them with those in the Brazilian population using estimates described by Senna et al.^27^ for RA and SLE, and by Horimoto et al.^28^ for SSc. We did not find studies with prevalence estimates for AAV in Brazil.

All statistical analyses were performed with the statistical package R, version 4.3.3 (R Development Core Team, Auckland, New Zealand).

## RESULTS

Between January of 1999 and December of 2023, a total of 423 patients were diagnosed with silicosis at our center. However, 54 patients were excluded from analysis due to the following reasons: 39 patients never came to any follow-up evaluation nor contributed to follow-up period; 13 patients had sarcoidosis and silicosis and required systemic glucocorticoid treatment; and 2 patients received kidney transplantation before starting follow-up and were under immunosuppressive therapy. The ARD group and the control group comprised 35 (9.5%) and 334 (90.5%) patients, respectively (Table 1). Compared with the control group, the ARD group had lower mean FVC% and lower mean TLC%. The remaining characteristics were similar in both groups (Table 1). Patients were followed for a median time of 3.8 years (IQR: 1.1-8.2).

**Table 1:** Group characteristics.^a^

Characteristic	Group	
	ARD	Control
	n = 35 (9.5%)	n = 334 (90.5%)
Female gender	2 (5.7%)	6 (1.8%)
Mean age, years	55.2 ± 9.6	52.7 ± 13.9
Tobacco use		
Never	16 (45.7)	160 (47.9)
Ever	16 (45.7)	168 (50.2)
No information	4 (11.4)	6 (1.8)
Diabetes	4 (11.1)	42 (9.3)
HIV	0 (0.0)	1 (0.3)
Median time of follow-up, years (IQR)	4.6 (2.0-11.3)	3.8 (1.1-7.5)
Median time of silica exposure, years (IQR)	15.0 (5.5-22.5)	16.0 (8.0-27.0)
Pulmonary function parameters		
Mean FVC%	64.7 ± 22.9	74.5 ± 21.2*
Mean FEV_1_%	58.3 ± 18.8	62.4 ± 24.8
Mean TLC%	77.2 ± 18.9	88.9 ± 23.8*
Mean DL_CO_%	58.1 ± 25.3	66.0 ± 28.7
FEV_1_% > 80% pred	5 (14.3)	77 (23.1)
80% > FEV_1_% > 50% pred	14 (40.0)	126 (37.7)
FEV_1_% < 50%	10 (28.6)	92 (27.5)

a Values are expressed as n (%), except where otherwise indicated. ARD: autoimmune rheumatic disease. *p < 0.05 in a two-tailed Student’s t-test.

### Autoimmune rheumatic diseases

Of the 35 patients in the ARD group, 12, 9, 6, 6, and 2 were diagnosed with RA, SSc, SLE, UCTD, and AAV, respectively (Table S2). Regarding their treatment, 24 patients (68.6%) received systemic glucocorticoids for more than six months; in addition, 21 (60.0%) and 5 (14.2%) patients received any csDMARD ever and any b/tsDMARD ever, respectively (Table S2).

Compared with Brazilian estimates, the PRs of RA, SLE, and SSc were 6.87 (95% CI: 3.20-14.74), 10.87 (95% CI: 2.44-48.38), and 225.49 (95% CI: 114.45-444.25), respectively.

### Risk of mycobacterial infections

Before starting follow-up, 55 patients reported having had a diagnosis of tuberculosis (4 and 51 in the ARD and control groups, respectively). We had no access to any AFB smear or culture results to check whether these diagnoses were accurate.

**Figure 1:**
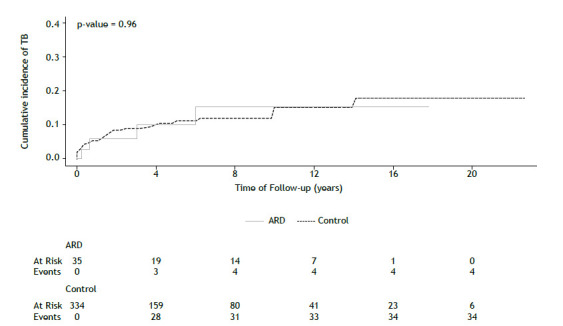
Kaplan-Meier curve of cumulative incidence of tuberculosis and number at risk in each group. TB: tuberculosis; and ARD: autoimmune rheumatic disease group.

**Figure 2:**
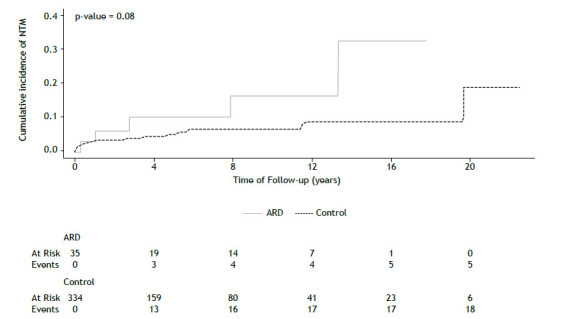
Kaplan-Meier curve of cumulative incidence of nontuberculous mycobacteria disease and number at risk in each group. NTM: nontuberculous mycobacteria disease; ARD: autoimmune rheumatic disease group.

During follow-up, 38 patients developed tuberculosis, 23 patients developed NTM, and 76 patients were diagnosed with latent tuberculosis (Table 2). All patients were referred to a tertiary care service for tuberculosis treatment. The cumulative incidence of mycobacterial infections did not differ between groups (Figures 1 and 2). We evaluated the univariate hazard risks of mycobacterial infections using the Cox proportional hazards model (Table 3). The risk of symptomatic tuberculosis was lower in patients who received treatment for latent tuberculosis during follow-up (hazard ratio [HR] = 0.34; 95% CI: 0.12-0.95). FVC% and FEV_1_% in higher quartiles were associated with a lower risk of NTM (HR = 0.43; 95% CI: 0.26-0.71; and HR = 0.46; 95% CI: 0.29-0.75, respectively).

**Table 2:** Prevalence of mycobacterial infections.

Mycobacterial Infections	Group	
	ARD	Control
Previous tuberculosis, n (%)	4 (11.4%)	51 (15.2%)
Latent tuberculosis, n (%)	8 (22.9%)	68 (20.4%)
Mycobacterial infection during follow-up, n (%)	7 (24.1%)	43 (12.9%)
*M. tuberculosis*	4	34
*M. avium*	1	1
*M. kansasii*	3	17
*M. smegmatis*	1	0

ARD: autoimmune rheumatic disease.

**Table 3:** Risk of mycobacterial infections. Unadjusted hazard ratio (95% CI) for selected variables.

Variable	Tuberculosis	NTM
Age > 60 years	0.86 (0.43-1.7)	0.74 (0.29-1.9)
Tobacco use ever	0.90 (0.47-1.7)	0.46 (0.19-1.1)
Latent tuberculosis treatment	0.34* (0.12-0.95)	0.54 (0.18-1.6)
Diabetes	0.91 (0.32-2.6)	0.70 (0.16-3.0)
FVC%^†^	0.82 (0.6-1.1)	0.43* (0.26-0.71)
FEV_1_%^†^	0.90 (0.66-1.2)	0.46* (0.29-0.75)
Time of exposure > 20 years	1.30 (0.64-2.5)	0.79 (0.32-1.9)
Any autoimmune rheumatic disease	0.97 (0.34-2.7)	2.40 (0.88-6.5)
Chronic glucocorticoid use	0.63 (0.15-2.6)	1.80 (0.53-6.1)
csDMARD therapy	0.36 (0.05-2.6)	2.10 (0.63-7.2)
b/tsDMARD therapy	1.9 (0.26-14.0)	3.0 (0.4-23.0)

NTM: nontuberculous mycobacteria disease; csDMARD: conventional synthetic disease-modifying antirheumatic drugs; and b/tsDMARD: biologic/targeted synthetic disease-modifying antirheumatic drugs. *p < 0.05 in a Wald test. ^†^per higher quartile.

The IR of tuberculosis and NTM in the overall cohort was 21.72 per 1.000 person-years (95% CI: 14.19-29.25) and 15.61 per 1.000 person-years (95% CI: 9.23-22.00), respectively. Compared with the adult Brazilian male population, the age-adjusted IRR of tuberculosis was 20.46 (95% CI: 14.89-28.13).

## DISCUSSION

In our 24-year cohort of patients with silicosis, having ARD did not affect the incidence of mycobacterial infections. Treatment of latent tuberculosis decreased the risk of symptomatic tuberculosis. Having higher FVC% and FEV_1_% were associated with a lower risk of NTM. To our knowledge, this is the first study to assess the incidence of mycobacterial disease in patients with silicosis and ARD. Previous studies mainly focused on ARD diagnosis.^2^ ^-^ ^5^ ^,^ ^10^ ^-^ ^13^

The prevalence of ARD in our cohort was 9.5%, in line with previous studies.^2^ ^,^ ^29^ ^-^ ^31^ Many recent silicosis registries reported high prevalence rates, ranging from 5% to 27%: the Michigan Surveillance System for Silicosis reported a prevalence of 5.5% (44/790) in a 1985-2006 cohort^2^; a Spanish study^29^ reported a prevalence of 11.0% (54/489); an Israeli cohort of artificial stone silicosis^30^ reported a prevalence of 22.5% (9/40); and a US case series of severe silicosis^31^ reported a prevalence of 27.7% (5/18). Epidemiological data have strengthened the association between silica and ARD, and experimental studies have yet to understand the mechanisms by which silica exposure affects immune regulation and triggers autoimmunity.^32^

The IR of tuberculosis in our cohort was 20-fold the incidence in the adult Brazilian male population. We chose to compare it with the IR in the adult male population, because there were few women in our cohort (8/369; 2.2%) and the IR of tuberculosis in Brazil is higher in males than in females: in 2023, 69.2% of new cases of tuberculosis occurred in males.^25^ ^,^ ^33^ The IR in our cohort was high, with rates comparable with a cohort of gold miners in South Africa in the 1990s, which reported an annual IR of 27 per 1,000 population in a sample with high HIV prevalence.^8^ The high IR of tuberculosis in our cohort may be explained by the high proportion of severe silicosis in our cohort (27.6% had FEV_1_% < 50%), as our hospital also hosts a national referral service of lung transplant. In this regard, it is possible that the IR of tuberculosis in this cohort is an overestimation of the true incidence of tuberculosis in individuals with silicosis in Brazil.

Diagnoses of NTM infections have been increasing in the past few decades worldwide due to multiple possible reasons, such as better laboratory techniques, increased tuberculosis/NTM awareness, ageing population, higher prevalence of chronic lung diseases, environmental/climatic changes, and immunosuppressive states.^34^ In individuals with ARD, the incidence and the prevalence rates of NTM are higher than in non-ARD individuals,^35^ ^,^ ^36^ and they may develop more severe NTM disease^37^ due to the immunosuppressive treatment. In our cohort, the incidence of NTM was similar in both groups, but with a trend towards being higher in the ARD group; a possible explanation was the lower mean FVC% in this group, which is equivalent to more severe lung disease.

Biologic/targeted synthetic treatment changed the treatment of several rheumatic diseases in the early 2000s, but it was associated with increased mycobacterial infections, especially in TNF-α-targeted therapy users.^10^ Because only 5 (14%) patients received b/tsDMARD therapy, we were unable to evaluate the risk of mycobacterial disease properly in these patients.

Our study had some limitations. First, we could not access patient data about previous mycobacterial infection diagnosis and treatment before commencing follow-up at our institution. As pulmonary tuberculosis and chronic silicosis have similar radiological features,^16^ some patients with silicosis might have been misdiagnosed as having tuberculosis if the diagnosis was solely based on radiological evaluation with negative sputum. Previous tuberculosis is a known risk for development of both NTM^7^ and recurrent tuberculosis.^38^ Second, because the ARD sample was small, the analysis was underpowered to detect a significant difference in the cumulative incidence of NTM. Third, few ARD patients used b/tsDMARD therapy, so we were unable to assess properly this subset of patients with high risk of mycobacterial infection. Finally, because we did not have a negative control group (i.e., individuals without silicosis), we resorted to comparing our data with general population estimates, which might have overestimated our results, especially considering that the estimates of RA, SLE, and SSc were based on small scale studies in Brazil,^27^ ^,^ ^28^ which may not reflect the actual prevalence of these diseases.

In conclusion, in our cohort of patients with silicosis, having ARD did not affect the incidence of mycobacterial infections. Prevalence rates of ARD were higher than in the general population, as was tuberculosis IR. Our findings strengthen the association between silicosis and ARD and further explore the risk of mycobacterial disease in these patients.
